# Exploring the role of leaders in enabling adaptive capacity in hospital teams – a multiple case study

**DOI:** 10.1186/s12913-022-08296-5

**Published:** 2022-07-13

**Authors:** Birte Fagerdal, Hilda Bø Lyng, Veslemøy Guise, Janet E. Anderson, Petter Lave Thornam, Siri Wiig

**Affiliations:** 1 SHARE – Centre for Resilience in Healthcare, Faculty of Health Sciences, University of Stavanger, N-4036 Stavanger, Norway; 2 Haukeland University Hospital, Bergen, N-5021 Norway; 3 Department of Anaesthesiology and Perioperative Medicine, The Alfred and Monash University, Melbourne, VIC 3004 Australia; 4 Haraldsplass Deaconess Hospital, N-5009 Bergen, Norway

**Keywords:** Resilience, Adaptive capacity, Hospital managers, Leaders, Teams, Organisations, Quality, Patient safety, Risk

## Abstract

**Background:**

Resilient healthcare research studies how healthcare systems and stakeholders adapt and cope with challenges and changes to enable high quality care. Team leaders are seen as central in coordinating clinical care, but research detailing their contributions in supporting adaptive capacity has been limited. This study aims to explore and describe how leaders enable adaptive capacity in hospital teams.

**Methods:**

This article reports from a multiple embedded case study in two Norwegian hospitals. A case was defined as one hospital containing four different types of teams in a hospital setting. Data collection used triangulation of observation and interviews with leaders, followed by a qualitative content analysis.

**Results:**

Leaders contribute in several ways to enhance their teams’ adaptive capacity. This study identified four key enablers; (1) building sufficient competence in the teams; (2) balancing workload, risk, and staff needs; (3) relational leadership; and (4) emphasising situational understanding and awareness through timely and relevant information.

**Conclusion:**

Team leaders are key actors in everyday healthcare systems and facilitate organisational resilience by supporting adaptive capacity in hospital teams. We have developed a new framework of key leadership enablers that need to be integrated into leadership activities and approaches along with a strong relational and contextual understanding.

## Background

“When the winds of change blow, some people build walls and others build windmills” Chinese proverb [1]

Resilience in healthcare can be defined as “the capacity to adapt to challenges and changes at different system levels, to maintain high quality care” [2] p.6) Resilient healthcare is a research tradition exploring how healthcare systems and stakeholders adapt and cope with a large range of variations to enable high quality care. Understanding adaptive capacity has been a key concern in resilient healthcare studies [3]. Adaptive capacity is the ability to adapt to external and internal demands by reframing, aligning, coping and innovating [4]. Research in this field has initially focused on theoretical development and concept building, with most research taking place in acute and emergency settings [5]. However, the literature lacks empirical studies testing the theories, as well as explorations of the underlying dynamics of resilience and adaptive capacity [6, 7]. As a result, there is limited knowledge of how systems, such as healthcare organisations, can achieve greater degrees of resilience. Further research applying empirical approaches such as case studies, longitudinal designs, multilevel and mixed method designs are needed to validate theoretical constructs and extend the evidence base [8]. Furthermore, we need research designs that address the complexity and adaptive capacity of healthcare systems as a whole [5] instead of focusing on the ability of individual system actors to act in resilient ways.

Resilience as a phenomenon is related to organisational responses to disruptions, turbulence, and discontinuities, and involves the ability to endure and adapt to new risk environments [8]. It is also related to how these systems succeed and are able to adapt and integrate new and innovative ways of working such as using technology and tools to improve quality in service provision [9]. Although the importance of resilience in everyday healthcare operations has gained increased attention in recent years [10] few studies have investigated the role of teams in providing adaptive capacity [3]. The increasing complexity of healthcare organisations makes it more and more necessary to rely on collaboration within and between professional groups to provide quality in care and treatment, consequently more work in hospitals is now team based [11, 12]. Teams are expected to increase adaptability, productivity, and creativity compared with what individual employees can offer on their own [13]. But our understanding of how teams coordinate their efforts to respond flexibly to emerging problems is unfortunately limited as is the role of leadership and organisational support for adaptive capacity in teams [3]. As there are gaps in our knowledge related to the team-based nature of adaptive capacity, there is a need to study different types of teams in different settings to understand how leadership and organisational structures support adaptive capacity [3].

 This study is part of the Norwegian contribution to the international study of Resilience in Healthcare [3], an international cross-country, multilevel comparative study of resilience in healthcare taking place in six countries. The Resilience in Healthcare study aims to add knowledge of how resilience is enabled in healthcare systems by examining adaptive capacity in different types of hospital teams. Four types of teams that differed in structure and purpose were included; (1) *Structural;* co located, uni/multi professional, prolonged teamworking, (2) *Hybrid;* some permanent and some rotating staff, planned teamwork, (3) *Responsive*; acute and unplanned episodes of teamwork, mobile teams (4) *Coordinating*; planned episodes of teamwork integrating representatives from multiple teams [14, 15]. The four teams are presented in Table 1.

**Table 1:** Team description of the four team types (Anderson et.al) [3]

Team type	Structural team	Hybrid team	Responsive team	Coordinating team
Location	Co located	Partly co located	Mobile	Meeting
Membership	Stable	Combination of stable and rotating	Varying	Stable
Ways of teamwork	Long term	Long term/planned episodes	Acute responses	Planned episodes
Affiliation	Ward team caring for patients	Ward team and rotating members	Team members who work in different departments	Ward leaders representing their departments
Examples	Ward teams	Acute admission units	Emergency response teams (e.g., stroke and cardiac arrest teams)	Capacity meeting across organisational units

Maintaining quality and safety is an ongoing challenge for hospital leaders whose units are tasked with delivering ever more specialised care under complex conditions, such as simultaneously managing acute admissions, staff shortages, and deteriorating patients [16]. Previous research has shown that leaders are vital for team effectiveness and improving quality and patient safety [17, 18], ensuring organisational resilience [19, 20] and managing the conflicting goals of safety and production demands [21]. However, there is a need to understand more about how the work of leaders is done to better understand, manage, and improve quality in healthcare settings, and thus understand how leaders influence adaptive capacity. Because research on organisational adaptive capacity has traditionally been conducted in fields other than leadership, the implications of leaders’ influence are not always clear [20]. This study seeks to close this knowledge gap by investigating adaptive capacity in teams through the lens of leadership and leaders’ own experience and practice. The aim of this study was to develop new knowledge about the role of leaders in supporting and enabling adaptive capacity in hospital teams. The following research question guided the study: In what ways do leaders enable adaptive capacity in hospital teams?

By investigating how these four team types are led and what leaders do to facilitate the teams’ adaptive capacity, this study contributes new knowledge and a new framework, both of which are important for future leadership practice and further theory development in our effort to conceptualize and operationalise resilient performance.

## Methods

### Design and setting

Due to little previous research on the role of hospital leaders in enabling adaptive capacity, a qualitative exploratory methodology was deemed appropriate. The study was designed as a multiple embedded case study conducted in two Norwegian hospitals [22]. A case was defined as one hospital containing four different types of teams. The following team types were selected and studied in each hospital according to the study protocol [3] (Table 2): *Structural teams*. Ward based whose members routinely worked together and comprised nurses and nursing assistant coordinating their actions to care for neuro and surgical patients. The *hybrid teams* had a mix of permanent and rotating members. These teams had a permanent nursing team and a rotating medical team. They were located at short stay units, such as emergency care and diagnostic short stay units. The *Responsive teams’* members were located at different departments and reacted to emergencies of cerebral infarction with time limited episodes of teamwork. The team had well defined aims and methods of working. The *Coordinating teams* facilitated decision making and workflow. Their work spanned hospital units, coordinating patient flow across the hospital.

**Table 2:** Description of the observed team and leaders in the study

Team type	Structural team		Hybrid team		Responsive team		Coordinating team	
Hospital	Hospital 1	Hospital 2	Hospital 1	Hospital 2	Hospital 1	Hospital 2	Hospital 1	Hospital 2
Name	Neurology ward team	Surgical ward team	Diagnostic ward team	Emergency ward team	Stroke responsive team	Stroke responsive team	Bed coordination team	Bed coordination team
Location	Co located bed ward team caring for neurology patients	Co located bed ward team caring for orthopaedic and surgical patients	Partly co located team in diagnostic short stay unit	Partly co located team in emergency ward	Emergency responsive cerebral stroke team	Emergency responsive cerebral stroke team	Bed capacity meeting across organisational units	Bed capacity meeting across organisational units
Professions included in the team	Nurse, auxiliary nurse, physician	Nurse, auxiliary nurse, physician	Nurse, auxiliary nurse, physician	Nurse, physician	Nurse, physician, radiotherapist	Nurse, physician, radiotherapist	Head Nurse	Head Nurse
Defined team leader role	Head nurse	Head nurse	Head nurse	Head nurse	Physician	Physician	Head nurse	Head nurse

### Recruitment of case hospitals and study context

The recruitment process followed the guidelines from the international study protocol of the RIH project, of which study is a part [3]. Both hospitals were selected and invited based on their different size and teaching role to ensure variability. Hospital 1 is a large University hospital and Hospital 2 is a medium size local hospital. The two hospitals are situated in the same health region. In the Norwegian health system,’ responsibility for health care provision is divided between municipalities who are responsible for primary care services, and the state which is responsible for specialised healthcare. Furthermore, the state has delegated and divided the responsibilities into four regional health authorities. Access to the hospitals was gained by contacting the respective hospitals’ research departments and hospital leaders who were part of the professional network of researcher BF. After presenting the project for the hospital’s leaders, we collaborated with the hospitals to select the different teams based on the recruitment specifications.

### Data collection

Data collection methods included observations, interviews, and document analysis. Data were collected between December 2019 and June 2020. The observations were undertaken by two researchers (BF and HBL) shadowing one or several team members during their workday. As the four teams differed in how they collaborate, the exact observation methods had to align with their work organisation. For the Structural and Hybrid teams the researchers followed one or several team members during an evening shift and the subsequent day shift for two consecutive workdays. For the Responsive teams the researchers shadowed one of the team members during their shift and followed them when they responded to acute alarms, enabling the researchers to be present during the acute episodes of teamwork in the Responsive teams. The Coordinating teams met for 10 to 15 min in a daily planned meeting. The researchers observed these meeting for a two-week period. In one of the hospitals this meeting was converted to an online meeting due to the Covid – 19 pandemics, consequently the researchers attended digitally alongside the other participants.

This resulted in a total of 115 h of observations as shown in Table 3. The researchers used an observation guide based on central concepts from the resilience literature and essential features of teams. These were team characteristics, team organisation, collaboration, and communication within the team, demands from the superior levels, structure (physical, technological, and other resources) and observable misalignments between demands and capacity. The Concept for Applying Resilience Engineering—(CARE) model [23], was used to collect and interpret the observational and interview data, including pressures experienced and adaptations made by team members and, their planned activities versus actual activities carried out. The CARE model is a guide to operationalize key concepts of resilience engineering, including the difference between work as imagined and work as done, misalignments between demand and capacity and the need for adaptations and adjustments to maintain acceptable outcomes. Accordingly, we looked for activities that could indicate how leaders responded to the need for adaptations and adjustments and enabled their teams to adapt, by, for example, conducting debriefs after incidents or reallocating resources. During observations, the researchers asked questions that were relevant to understand everyday work and how adaptive capacity was enabled and supported. After each observation the researchers wrote individual observation notes using the observation guide to structure the text. In this article we used the observation material related to the leaders’ role in enabling adaptive capacity.

**Table 3:** Overview of observation and interviews

**Hospital 1**		**Hospital 2**	
**Team**	**Interviews**	**Team**	**Interviews**
Structural	1	Structural	1
Hybrid	1	Hybrid	1
Responsive	1	Responsive	1
Coordinating	3	Coordinating	4
**Total interviews**	**6**	**Total interviews**	**7**
**Total observation**	**52 h**	**Total observation**	**63 h**

Interviews with Coordinating team members and team leaders from Structural, Hybrid and Responsive teams were conducted post observation using semi structured interview guides. As such the interviews could elaborate on findings from the observations. Researcher BF made the interview appointments with each team member or leader for the weeks following the observations. Two slightly different interview guides were used in this study, one for the interviews with team leaders and one for the interviews with team members, to better frame the perspectives. The themes covered in the two guides had similarities, such as opinions and experiences of teamwork; the organisation of teams; descriptions of collaboration and communication with and within the teams; experiences of demands from superiors both within the team and the broader organisations; and experiences of dealing with misalignments between capacity and demands. In addition, the interview guide for team leaders also focused on their leadership role in the organisation.

Interviews were conducted with thirteen leaders. Eight of the interviews were conducted with each of the leaders of the eight different teams included in the study: Structural, Hybrid, Responsive and Coordinating teams, using the interview guide for leaders. In addition, five interviews were conducted with members of the Coordinating teams, i.e., the bed allocating teams consisting of leaders from all the wards in both hospitals, who met daily to discuss bed capacity. When these leaders were interviewed in their capacity as members of the Coordinating team, they also spoke about their role as team leaders on their respective wards. We therefore included those parts of the interviews with these five participants in our material on leaders, as it brought richness to the overall data while aligning with the aim and research question of this article (See Table 4 for detailed information on the interview’ participants). Researcher BF conducted all the interviews. All interviews were audio recorded. Most of the interviews were held face to face at the leader’s workplace, but due to Covid-19 restrictions three of the interviews were conducted digitally. The length of interviews varied from 45 to 90 min, with a median length of 70 min. Transcribed interviews and observation notes totalled 209 pages.

**Table 4:** Characteristics of the interviewed participants

Hospital 1				Hospital 2			
**Team**	**Profession**	**Sex**	**Age**	**Team**	**Profession**	**Sex**	**Age**
**Structural**	RN^a^	F	44	**Structural**	RN	F	59
**Hybrid**	RN, MSc^a^	F	40	**Hybrid**	RN	F	56
**Responsive**	Physician, PhD^a^	F	43	**Responsive**	Physician	F	43
**Coordinating**	RN	F	39	**Coordinating**	RN	M	33
	RN	F	47		RN	F	37
	RN, MSc	F	47		RN, MSc	F	43
					RN	F	56
**Sum**	RN = 5 Physician = 1	F = 6			RN = 6 Physician = 1	F = 6 M = 1	
**Interviews** ***N*** **= 13**							

^a^*RN* *Registered nurse. MSc* *master’s degree in healthcare leadership, PhD* *Doctoral degree in medicine*

### Analysis

All interviews were audio recorded and transcribed verbatim by researcher BF. Observation notes and interviews for each team at each hospital were grouped to facilitate the analysis. The analysis was performed with an inductive approach. We conducted a qualitative content analysis of data material according to Graneheim and Lundman [24]. Qualitative content analysis offers the opportunity to analyse the manifest content as well as the latent and interpretative content of the collected data [24].

Interviews, transcripts, and observation notes were read through several times by all authors to achieve a sense of the whole, followed by discussions in the research group. In the first round of analysis all transcribed interviews were individually searched for meaning units, which were then discussed with all members in the research team. Secondly, the meaning units were condensed and divided into tentative codes. The codes and their interrelations were discussed several times in the research group and sorted into subcategories, categories, and themes. Observation notes were included in the analysis and coded using the same approach. We found that the observation notes correlated with what the informants talked about in the interviews. The analytic process moved back and forth between the data and the themes, resulting in four themes representing the activities engaged in by hospital team leaders to enable adaptive capacity, called enablers. Table 5 shows an example of the analytic process.

**Table 5:** Example of qualitative content analysis process

**Quote**	**Condensed meaning unit**	**Codes **Manifest	**Subcategory**	**Category**	**Themes **Latent
**Every other Tuesday we have simulation. Two of the nurses get a case, and then they are going to do the whole chain if they have to call a physician or if they have to call intensive care. It's very educational. Not everyone thinks it's great fun, but they're getting better and better**	Fixed day for simulation training, educational even if some thinks is unpleasant	Fixed day for simulation, deciding who is attending	Arrange simulation training as part of competence development	Facilitate simulation/practice-based learning	Building competence in the teams
**It is difficult and there are a lot of considerations to be done. Putting together the team that you think works well or this doesn't work, right? So, I feel a lot of times that I spend an enormous amount of time on distribution team members. Because I kind of distribute to the evening shift and to the night shift and do a rough distribution of the morning shift the next day. And then you often put a couple of positions open and so that you redistribute, because a lot can happen during the evening and nightshifts**	Difficult to disperse employees to the teams considering multiple factors. And the workload situation changes during the different shifts. Time-consuming activity	Multiple factors to consider when putting together the teams, considering what will work well and what will not	Considering multiple factors when composing the teams. Time-consuming and ongoing task	Risk based staffing	Balancing competence, workload, risk, and staff needs
**A low functioning team is if you as a person is somewhat rigid, and don’t want to change or adjust to the ward’s needs. Or not wanting to help out, because we are dependent on that. Otherwise, it will not work**	The ward is dependent on employees who are willing to help each other and adjust to requests	Dependent on flexible co-workers adjusting to the wards needs	Dependent on workers to be a good colleague and build relationships	Collegial support and flexibility	Relational leadership
**Evening shifts were reported very busy, and our software also confirmed that the income of patient was at its peak in early afternoon, and so we altered one of the day shifts to a middle shift to match the staff amount with patient amount. This was a new shift with working hours from 11 am. to 18 pm. It has been a success**	Busy evening shift due to high income of patient in early afternoon led to altering the shift plan and creating a new role/working hour to match the staff amount with the patient amount	Busy evenings shift due to high income of patient led to change in work schedule for better match of patients and staff	Busy evening shifts led to changing the shift plan in to staff in line with the number of patients	Changing the working schedule to match the needs of the workplace	Situational understanding of work practice needs

### Results

**Fig. 1:**
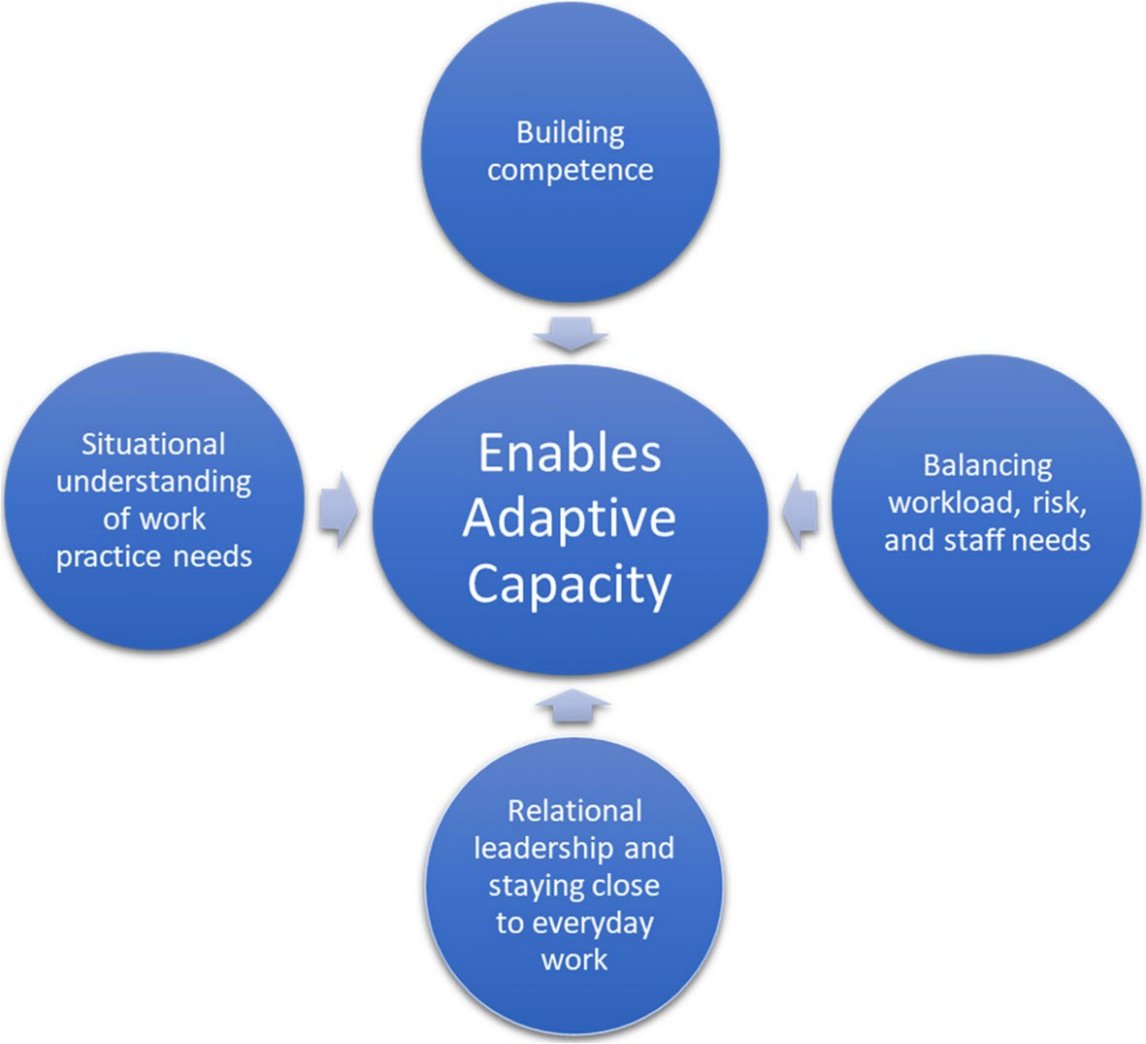
Four enablers for adaptive capacities in teams

The results showed that team leaders engaged in four enabling activities to support the adaptive capacity of their teams. As shown in Fig. 1, the four themes were (1) Building competence, (2) Balancing workload, risk and staff needs, (3) Relational leadership – staying close to everyday work and (4) Situational understanding of work practice needs. In the following sections we describe these enablers and activities enacted by the leaders.

#### Building competence in the teams

##### Facilitating training and education for new employees

The leaders emphasised that healthcare has increasingly become more and more specialized, which led to a need for an increased specialized competence among the employees. Leaders, across all teams, focused on their own role in facilitating adequate training and professional skills development among the team members. In general, new employees rely on considerable tuition and training in the first months of their employment to become competent at their job and function on a secure professional level. The hospitals included in this study, had developed an induction program for the first weeks of employment, containing apprenticeship, specific procedures, guidelines and getting to know the environment of the workplace. Close follow up from the leader with regularly planned dialogue was a key part of the induction process. The competence plan developed in the wards estimates that it takes one year of ascending the steps from novice to competent worker. Correspondingly, new employees needed a lot of support to feel safe in their new role, and the leader had to pay attention to how the new employee was settling in and adjust the level of support provided as their needs changed. Responsive team members do not work together on a normal basis, and these teams depend on each team member already knowing their role. The leaders therefore paid extra attention to team members’ training through simulation activities and ensured that newcomers acted as observers within the team prior to being given responsibilities themselves.

“They get tuition and the written procedure before they join the team as an observer and perform under supervision before they are on their own” (Leader of responsive team hospital 1).

##### Monitoring competence among staff

The leaders pinpointed that they depend on having competent staff and are continually working to facilitate the team members competence development. It was therefore important that leaders monitor the competence among the staff as part of ensuring the safety and quality of the service provision. All leaders in the study argued they were familiar with the competence level of each individual employee. Some leaders explained the use of formal systems for monitoring the competence level through software programs where the employees register their courses, certifications, and training attendance. In some teams there were defined competence levels for specific team roles, e.g., shift leader, and leaders used this information about competence levels when staffing the different shifts. The leaders also made sure that employees are offered the training and courses they need and have annual recertifications rounds which were documented in the software used.

“… we have that software where we add all the things they need to know. They have to follow this up themselves, but we monitor to see what they need…” (Leader hybrid team hospital 1).

##### Facilitating simulation and practice- based learning

For team members to be able to elaborate and maintain their competence as a key dimension for adaptive capacity, leaders highlighted that they need to facilitate competence development at the workplace for it to take place. Learning in the contextual setting was seen as being valuable and simulation-based training was one of the methods used for this purpose. Our observations of simulation training sessions in some of the teams we followed confirmed this.

All the leaders emphasised simulation as a valuable method for competence development. While not all teams had established well-functioning simulation groups, all the leaders talked warmly of the effect simulation has on their staff’s competence and their ability to handle a variety of everyday situational demands. While not all team members were positive about the simulation method, according to the leaders, most of them did acknowledge that this was a very productive method for learning. Especially for the responsive teams, simulation was of high value since this was the only way, apart from observation, that the team could practise their skills together and develop their collaboration capabilities. The leaders explained how they arranged simulation sessions on a regular basis and made sure that all employees participated. Since the responsive team members only work together during acute episodes and the teams are composed of members from different departments in the hospital, one of the key learning points in the simulation sessions for these teams was focused on communication and collaboration with team members they did not know well. This was most visible in the large hospital, while in the midsize hospital the Responsive team members were more likely to know each other. For instance, during an observation of the Responsive team in the midsize hospital, the team had performed simulation training with debriefs, and just one hour later the same team responded to an acute situation and managed to make use of several learning points they had discussed during the simulation session.

“…What we focus on in simulation is precisely good team leadership and communication. So, we practice less on the actual stroke course than team collaboration” (Leader responsive team hospital 1).

The leaders were aware of the role of reflection in practice as an important way for enabling adaptive capacity in teams. For example, reflection sessions after adverse events were used by leaders to debrief employees and encourage discussion. Some leaders also used debriefs to focus on aspects and adaptations that went well in the situation allowing for reflection of what they should do more of in their work. For example, during covid some of the employees ensured equipment availability by developing a kit containing all the necessary equipment for examination of new incoming acute patients with unclear Covid – 19 statuses. This adaptation enabled faster examination without contaminating unnecessary equipment or needing assistance from colleagues outside the room. The leader subsequently implemented this kit for all incoming patients with contagion risk.

##### Scheduling training to build competence

Our study found that the leaders emphasised the need for organizing tuition and training within workhours to enable teams’ adaptive capacity. Expanding their employee’s competence is important both for the safety of the patients but also to comply with governmental and other regulatory requirements, leaders said. Instruction guidelines of patient treatment from regulatory authorities are increasing, with the consequence that much of the work needs to be continuously aligned with these guidelines.

Hospitals are obliged to arrange competence development for staff both to train new skills and retrain certain skills, e.g., medication management. To manage these demands, the leaders planned for competence building as part of the staff’s long-term work schedules, by rostering compulsory training days alongside regular shifts. In addition, new or revised procedures need to be shared and updated amongst the team members as a prerequisite for safe work practice. Leaders responded to these demands by arranging fixed days of the week for tuition or information meetings throughout the year. Due to high workload on the ward, however, it was often difficult for staff to leave their regular work tasks to attend these pre-arranged meetings as there was not always a substitute staff member to take over responsibility for the patients. Another way of refreshing or building competence and buffer capacity was therefore by personnel rotation on different teams in the inpatient wards (structural and hybrid teams). In that way team members could learn new procedures, diagnoses, or refresh previously acquired competence.

*“…then you have the rotation of the teams. When a team member thinks that it has been a long time since they worked on the green team, they want to work there to refresh their knowledge and feel safe with the routines of the team again” (Leader structural team hospital 1).*

#### Balancing workload, risk, and staff needs

##### Facilitating careful mix of experience and skills within the teams

The leader’s effort in composing the teams to match the competence and capacity needed was an endless and time-consuming task. Both the amount of work, and the competence needed to complete work task varied significantly throughout the day. When adapting and managing team composition the leaders first assigned the tasks that required specialized competence, then they carefully distributed the more experienced team members to secure competence in every team. And finally they allocated the rest of the team members, while trying to also take into consideration the employee’s own needs and wishes. It was seen as crucial that every team included experienced team members for the teams to function optimally. During our observation we noticed that experienced team members shared knowledge and skills with the novices, leading them to enhance their competence. Furthermore, leaders informed us that some people functioned better together than others depending on personalities, relations, and motivation. Above all the leaders said that willingness among the team members to collaborate and help each other was of great importance because the team’s adaptive capacity relied on members’ collaboration to make the system work.

*“Otherwise, it doesn’t work … we don’t choose our colleagues and we don’t have to be best friends … but everyone has to contribute in order to make it a good day at work.” (Leader structural team hospital 2).*

##### Combining long term planning and aligning to the situation

Most of the leaders made 52-weeks shift plans for their teams. They explained how they took a lot of time to adapt the team’s competence mix, while at the same time trying to comply with individual team member’s needs. The leaders also needed to take the different trade union demands for healthy shift plans into consideration. They mostly experienced that this long-term shift plan got altered, due to employees leaving for different reasons or going on sick leave. So, despite the enormous work undertaken with this long-term planning effort, adaptive short-term planning was always necessary as well.

During the observations we noticed the change in teams’ capacity with deteriorating patients or acute admissions leading to shifting more resources from one team to another. When the teams were not able to cope with a situation, they asked the leader for help with either prioritizing tasks or getting more resources. This could also imply an adaptation by the leader to organize extra resources or reallocate resources for the next shifts.

*“… I plan for the evening and night shift, and then I roughly plan the next day shift, leaving a couple of positions open for redistribution the next morning, because a lot can happen during the evening and night shifts.” (Leader structural team hospital 1).*

##### Staffing according to risk and experience

The leaders worked to align resources and demands to reduce patient risk. Teams that had extra responsibilities or functions outside the team, e.g., also having responsibility in the responsive team, were allocated extra personnel to make them able to cover for staff being busy with call outs. This adaptation from the manager provided a buffer for the team when a team member left the team for acute episodes. The leaders carefully distributed key responsibilities to employees they knew could manage these tasks. Placing competent and experienced employees in key positions was a way of securing capacity for managing risk.

*“You have the numbers in your head, who is laying in the beds, how many need constant observation, how many need monitoring …basically the number of resources needed. And then you look at who is coming on the shift, distribute after competence and all the extra responsibilities. It is a lot to distribute and quite difficult at times” (Leader structural team hospital 1).*

When an experienced person on sick leave from one of the teams needed to be replaced with another experienced staff member, the replacement staff member often moved from another team. For example, during our observation of a morning hand-over, one employee called in sick and the leader then had to move an experienced nurse from another team and replace that nurse with a less experienced nurse.

## Relational leadership and staying close to everyday work

### Enacting collegial support and flexibility

The leaders emphasised how they engaged with staff to create a culture of involvement, caring, and helping each other. They spoke of having a culture characterized by support and flexibility where willingness to help each other in peak situations improved the overall capacity of the team. When hiring new employees, the leaders looked for good team players. Good relations amongst the team members were seen as important for the team’s capacity to adapt.

*“To be able to help each other, take over tasks, play ball with, discuss professional matters, discuss difficult cases, next of kin, like… I think that is extremely important” (Leader hybrid team hospital 2).*

By creating good relations between the team members, the team members got to know each other’s strong and weak sides and personal preferences. Leaders argued that it was easier to distribute tasks amongst team members, and for them to trust each other, if they already had developed good relations. During an observation of the structural team, the team members said that they sometimes preferred having fewer team members on a shift, rather than working with a substitute who was not familiar with the team. It was more effective working with colleagues they knew compared to people they did not have any personal relationship with or knowledge of.

### Building relations and collecting information at the front line

Leaders, especially of the structural and hybrid teams, focused on the importance of knowing their employees, and they prioritized spending a lot of their workday close to the teams and team members, getting to know their strengths and weaknesses. They talked about the importance of signalling that they were available for the employees if they needed to talk to them and showed an interest in their personal lives. One of the leaders came to work one hour earlier than scheduled every morning just to be able to talk to the employees who had been on the nightshift. Apart from getting to know their employees the leaders also wanted to stay close both to the teams and the clinical context to get the correct information on the status of the ward. They felt that the software provided for monitoring patients’ conditions, staffing and workload indicators on the wards, did not display the whole story. For example, the monitoring software only counts the number of patients but not how much care each patient needs, which is highly important information for the teams, and which could change quickly. Consequently, the leaders felt that they had to continuously talk to the teams to get an overview of the status of the situation, real patient load, and where the situation was heading. The leaders then used this information to plan the next shifts.

By staying close to the team and the everyday work, the leaders knew what the employees experienced at work and in their personal lives. Furthermore, the leaders included feedback from employees of their individual needs and wishes when they planned the staffing of the shifts.

*“… and then we also look at which nurse is coming, right. And then we see that, okay she has had a couple of though shifts lately so she needs to get an easy room… we know much about the history of the employees, it can relate to illness, a bad back or just the need for some adaption of the tasks” (Leader hybrid team hospital 1).*

The leaders also had to make difficult decisions to avoid conflicts, for example, some leaders highlighted a specific person who would have to go and help a different team in case of increased work pressure. This was a way to mitigate possible future discussions and conflicts. The leaders also contributed themselves in peak situations to the manual work within the team, helping with tasks, or helping to prioritize. On some wards this was a regular part of the procedure for peak situations. As such, leaders tried to be close to the situational context.

### Engaging by building a culture of empowerment, self – organization and positive feedback

We observed that the teams were good at self-organization and coming up with suitable solutions by themselves when they needed to adapt their work practice. They always tried to manage with the resources available within the team before they asked other teams for help, and if that did not work, they involved their leaders. The leaders also pointed out that they encouraged the employees to identify solutions on their own. Moreover, the results showed that, if employees suggested a type of improvement, the leaders wanted to involve the employees in improvement work. The leader talked a lot about getting the employees committed and engaged in their work and surrounding environment as an enabling factor for adaptive capacity in the teams.

*“…great, that was a very good suggestion, can you do the work on that”. I often delegate improvement task to the employees, leading them to take ownership to the process. I think it is important” (Leader hybrid team hospital 1).*

During interviews the team leaders talked about focusing on the positive. They were conscious of balancing communication with positive feedback, and not only informing staff about adverse events, new rules, or regulations. When the leaders received positive feedback from patients or next of kin, they made sure to communicate this to the employees. Also, when the leaders heard about situations where an employee had successfully handled a situation or adapted to patient needs, they tried to give positive feedback to the employee afterwards. The leaders argued that this was strongly related to building a positive culture.

*“I try to communicate positive incidents of cooperation from yesterday in the daily meetings. If a ward has taken on extra patients and been very cooperative. Because it’s all about building culture” (Leader coordinating team hospital 1).*

In the Coordinating team leaders tried to facilitate involvement by bringing in the patient’s perspective when allocating patients to the different wards. Creating a culture of patient centredness as well as getting different healthcare professionals committed to a solution in the daily meetings, enabled interdisciplinary collaboration.

## Situational understanding of work practice

### Ensuring planning and monitoring of appropriate equipment and workload

Creating surroundings where work can be seamless and efficient is vital for the team’s ability to adapt. The leaders interviewed worked on both securing enough equipment and wanting to have software that better fitted the context. Financial constraints were always in the leader’s mind where they focused on balancing the need for more or better equipment, and more human resources at the frontline within their budgetary constraints. By trying to secure resources for their teams, leaders often negotiated with other departments to get hold of extra resources, balancing economics with patient safety.

*“…you always want more resources, but you always have the economy in the back of your head. You don’t bring in extra staff unless it is necessary, and you always try to arrange for the most economical solution” (Leader hybrid team hospital 2).*

The results showed that the team leaders monitored the patient numbers and corresponding workload during the day, to be able to adjust and plan for the next shift. The intention was to enable the staff off duty to rest and not take on extra hours. The leaders tried to have situational awareness for both everyday work and peak situations. They focused on aligning practices to national guidelines, adapting work practices to risk, and understanding the situational risk.

### Enabling capacity to anticipate and enacting a risk- based response

The leaders talked about trying to develop team members capacity to anticipate by engaging them in preparation for patient admissions. They tried to get ahead of events by continually monitoring the frontline situation.

*“I have spent a lot of time trying to get them to plan for acute admissions. Get them to acknowledge that they normally get four acute admissions pr. day and to plan for that” (Leader coordinating team hospital 1).*

As situations could change unpredictably, leaders had to make plans for peak situations. There was no budget for slack in staffing levels. On public holidays they often relied on their experience and data from previous years when staffing the different shifts. When they had made a specific plan, they ensured spreading of this information across shifts, so that employees knew what they had planned and could be prepared.

### Understanding everyday work

The leaders thought that the most important aspects for patient and staff safety were to be well prepared for normal situations. And that they needed to recognise the continuum of normal activity in their ward. Understanding every day work made it easier to adapt to peak situations. Having a plan for acute admission patients and knowing what to expect provided preparedness. Leaders also used their experience in risk-based planning and talked about the importance of having a holistic comprehension of the contextual situation and conditions. Knowledge of the overall context was crucial for leaders to make good judgements and adaptations.

“…we are depending on others, and you need to find where the weak link that led us to not accomplish our goal is” (Leader structural team hospital 2).

## Discussion

**Fig. 2:**
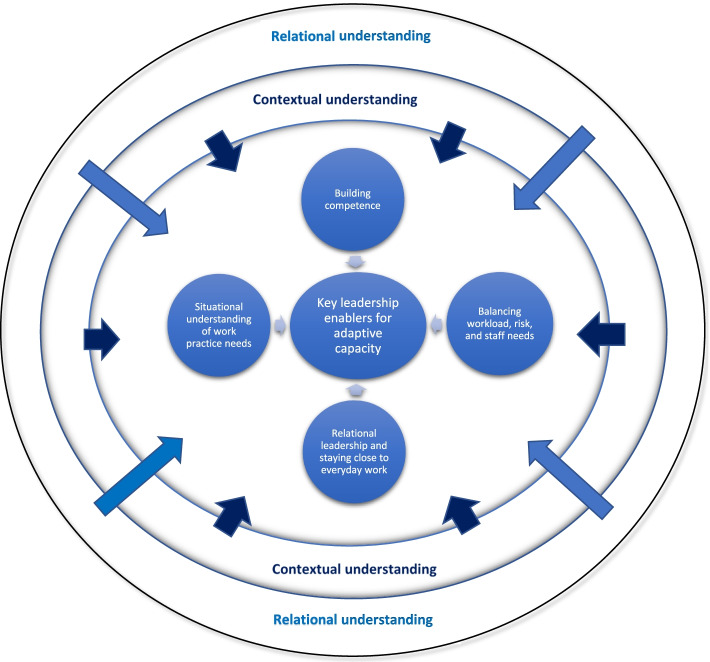
Framework for leadership enablers for adaptive capacities in teams

In this study we have explored the role of leaders in supporting and enabling adaptive capacity in teams. Through observation of four different team types and interviews with team leaders in two different Norwegian hospitals we identified the key enablers used by team leaders to support team adaptive capacity. The results show how the team leader role encompasses and balances all the four enablers of adaptive capacity in the teams, revealing the leader role as complex and multifaceted. Although the different teams had different challenges, overall, our study showed similar reflections and leadership practices focused on relational and contextual understanding as key dimension for enabling adaptive capacity in teams. Figure 2 illustrates by the use of two circles how this holistic approach, (including the four enablers, context, relation) must be integral to leaders’ decision making and approaches. Consequently, in the following, we discuss how a leadership role for adaptive capacity in teams is enacted in two ways; (1) leading through contextual understanding and (2) leading through relational understanding.

### Leading through contextual understanding

The study showed that a considerable amount of a leader’s everyday work was characterized by actively organising and reorganising to support and sustain the team’s adaptive capacity. In line with Lombardi et al. [25], we found that leaders guide the team towards a purpose, encourage development of the team and create engagement and commitment. The results demonstrated that to balance the workload so that the team could have a buffer for adaptive capacity, leaders continuously used their professional ethos, staff expertise and deep contextual knowledge. Here our study echoes the work of Hybinette et al. [16] who found that everyday work by managers in hospitals was characterised by actively organising and reorganising around the teams to support them. The ability to anticipate, resist and respond to adversity depends on leaders’ knowledge of adversity triggers in their workplace and within the organisation and its surroundings. Hence context specific knowledge and understanding of the setting, staff challenges, patient and care load, risks, complexity, and work processes are crucial for leaders [16, 20, 26]. Our study gave insight into how the team leaders must be able to notice changes in the risk profile of current situations (e.g., patient number, patient load, staff competence, experience, peak situations) and recognize that the limit of safety is about to be broken. This again requires knowledge of the organisational environment as well as risk sensitivity and sensemaking skills [8, 21]. Our study showed that team leaders possess a large network spanning organisational units and levels, enabling leaders to help teams in finding solutions to problems. In a smaller hospital, like Hospital 2 in our study, the distance to the top management is shorter than in a larger hospital (Hospital 1), and the participants in different positions are more likely to know each other than in larger hospitals. In our study,—being able to draw on relationships, networks and contacts appeared as important facilitators of leaders’ ability to promote adaptive capacity in teams. Further studies could investigate if and how team leaders adapt their leadership practice whether in large or small organisations.

Leading through contextual understanding was -illustrated by responsive team leaders who used their contextual understanding as input for deciding when to contact back-up experts to evaluate the safe treatment of the patient. Furthermore, leaders of structural and hybrid teams all emphasized the challenge of planning ahead due to an inherent situational complexity. However, these leaders sought to mitigate this uncertainty as much as possible by using the contextual experience when composing the team, anticipating activity on weekends and public holidays, and planning for everyday peak situations (e.g., Friday afternoons) as well as for extra-ordinary situations (e.g., admission of Covid-19 patients). Leaders of coordinating teams often found it necessary to visit the various wards in person to obtain an overview of the contextual situation in difficult decisions of reallocating of patients.

Situational awareness encompasses gathering information, making sense of it, and anticipating how the present situation may develop [11, 21]. Our study showed that leaders often experienced that there were limitations to what information they could get out of the organisation’s technical systems, hampering its accuracy for decision making. Based on the information they acquired through observing work and speaking with staff, they adapted and used their experience to monitor and anticipate overall workload, allowing them to continuously optimize and reallocate resources. Having an adaptive and proactive mindset enabled the leaders to rapidly recognize room for manoeuvre [27], which they found crucial for supporting adaptive capacity in their respective teams.

Our study showed that leaders tried to obtain two perspectives in their daily work. First the umbrella perspective of the overall situation and future needs (long term perspective and planning), and second, to be aware of the immediate ongoing situation (short term planning and response), taking into account all facets in their decision-makings, and reorganising to support adaptive capacity in teams. However, leaders were clear about the consequences of common trade-offs, such as sacrificing scheduled competence development for staff to respond to acute patient situations or a sudden and unexpectedly high flow of incoming patients [28–30]. Such trade-offs reduced the overall resilience of the teams in the long-term and was a constant struggle for the team leaders to manage. This corresponds with the work of Hybinette et al. [16] who found that each decision of sacrificing for example staff or patient education has implications for the organisation’s future capacity for resilience. Leaders interviewed in our study remained strong in their advocacy of the fact that to manage professional development, they have to know the teams and also equip them to manage everyday work. Building competence was important for resilience in the long term and this balancing act was highly related to how leaders were able to combine professional competence and context specific competence in the teams.

### Leading through relational understanding

Leaders’ behaviour has been shown to affect the internal dynamics of teams and influence team climate and learning orientation [31, 32]. Our study showed that leaders participated in everyday work by consulting the team, using shared decision making, and delegating responsibility to the team members. Previous research has found how team members are sensitive to leaders’ conduct, and study leaders’ actions to understand what is expected and acceptable in team interactions [31]. Our study showed that leaders were mindful of this and adjusted their behaviour accordingly. For example, by helping team members with their work tasks in peak situations and by including team members individual needs and wishes when planning and organising work. They also maintained a positive and inclusive manners towards all team members.

When teams adapt to meet an actual situation, new learning experiences and ways of operating emerge. For example, new routines emerged based on Covid -19 adaptations in all the teams in this study necessitating improved and innovative work practices. By staying close to the teams, leaders capture this and can transfer and integrate it in the organisation [33]. Our study showed that leaders in all teams tried to create a learning culture in which individuals could speak openly. A key feature of their relational approach to leadership, was an emphasis on building good relations with the team members, getting to know them on a more personal level to understand their work and home balance, and actively creating a climate with collective commitment to support and help each other. They also allowed the teams to individually adapt to circumstances to permit them to be part of a process of identifying conflicting goals in a complex environment [16], empowering the teams to find solutions of their own. In the literature, such empowerment of teams and including team members in decision making, is argued to nurture resilience in organisations [19, 34].

Correspondingly, by engaging employees in sharing their experiences, team leaders enhance the collaborative capability in the organisation [33]. Leaders’ inclusiveness affects psychological safety relationships, as stated by Nembhard and Edmondson [31, 35, 36]. In our study, leaders felt that by walking around, helping in pressing situations, and having an open-door policy, they were signalling that they were available for their team members. To get the best out of their team members, leaders argued that they had to know their employees and take care of each employee on an individual basis. This was achieved through being present at the front-line, coming in to work before the start of the morning shift to get to know the team members on the night shift, arranging communal Friday lunches, joining patient rounds, and by distributing and rotating demanding tasks (like working in isolation areas) among the team members to ease and equalise the burden. This is a vast task to take on, as leaders for the Structural and Hybrid teams interviewed in this study had responsibility for between 40 – 80 persons, which is similar to a medium to large company in Norway. The team leader’s ability to build relations and create psychological safety with the team members affect the team overall performance [32, 37]. Based on our results, future studies, and interventions to translate resilience and build adaptive capacity into practice need to acknowledge these characteristics of the team leader’s role.

In addition, creating an overall environment of mutual caring and trust was important for the leaders in our study. Leaders’ relational work included giving feedback, creating common reflexive spaces [38] and being available for the team members, all of which contributed positively to the team members behaviour towards one another and the culture on the ward. This is in line with previous research that emphasizes what is called the systems’ soft elements (skills, knowledge, decision-making processes, values, norms, relationships, and communication practices) as important for nurturing health system resilience [39, 40]. However, for those who had received little training in leadership and managerial roles, this knowledge was however gained through their own experience or by emulating others.

Our study also showed that leaders experienced that managerial task previously done by people were increasingly being replaced with computer software solutions that they then had to learn. Consequently, in our study, the bed capacity meeting (Coordinating team) was used to discuss challenges and functioned as an arena for debrief and support. This was particularly evident in Hospital 2 where participants at the meeting were fewer due to the size of the hospital. This may suggest that leaders need dedicated arenas for support and backing in their job as a key dimension for them to better enable adaptive capacity in their teams [25, 33, 39].

## Implications for practice

Our study found four key leadership enablers for adaptive capacity in teams. While the constant day to day problem solving and adaptations illustrated in our study are essential for safe service provision [9], they also potentially reduce resources available for the enabling activities that are essential for team adaptive capacity. These activities can be invisible, and their importance overlooked, but this study shows how the work of enabling adaptive capacity is essential for well—functioning teams. Articulating the value of this work and ensuring that it is resourced and acknowledged is important. The findings also have relevance for training and skill development opportunities for team leaders, by showing the importance of these enabling activities. Leadership significantly shapes the direction and organisation of the teams, and leadership enablers for adaptive capacity and developing the relational and contextual understanding demonstrated in the framework (Fig. 2) is imperative. Healthcare organisations should consider integration of a stronger emphasis on the importance of the leadership role for adaptive capacity in teams in both leadership training programs and in team processes, as part of a systemic improvement and not as an individual skill. Team leaders need to receive proper training to create a climate of psychological safety [13] and to have the opportunity to work with relational leadership. The importance of relationships should also be considered in the healthcare education system, focusing more on training in team work to better position students for their working life in healthcare.

## Limitations

This study was able to observe four teams in two hospitals during the Covid—19 pandemic. The observation was supported by interviews to understand how leaders worked. The data collection took place over 6 months and mapped how leaders of different teams used similar strategies to enable adaptive capacity in their respective teams. One might expect larger difference in leadership approaches based on the varying compositions and areas of responsibility of the four different team types in one large university hospital and one smaller hospital. Further research could however conduct deeper investigations into leadership practices through longer observation periods of the leaders themselves, as opposed to the teams as a whole to gauge potential differences. It is a limitation of our study that we did not observe all team leaders directly by following them specifically during their workday. Apart from observation of the coordinating team, which consist of leaders participating in the bed allocating meeting, our study mostly observed leaders indirectly while shadowing the team members and talking to them prior to and during these observations of the teams.

It could be considered a limitation that data collection took place during the initial waves of the Covid -19 pandemic in Norway, which constituted a time of exceptional pressure on health care system worldwide. However, this was arguably an ideal time to study resilience in healthcare systems and to observe how teams adapted to the increased demands they experienced. Our data constitutes a combination of interviews and observations which provided us with a rich material to understand our research problem [41, 42].

## Conclusion

In this study we have demonstrated that team leaders are key actors in everyday healthcare systems and central for organisational resilience by enabling adaptive capacity in hospital teams through relational and context sensitive leadership approaches. Leaders have a fundamental role in balancing control and adaptation in the everyday work of teams. The leaders’ role means they must develop different perspectives, including building good relations and understand the team, promoting psychological safety, and learning from team, meet the demands of the immediate situation, plan longer term resourcing, and handle trade-off decisions for the teams. This is the core of resilient leadership [16]. However, findings show that leaders receive insufficient formal training and support in their role leading them to use their professional network in the hospital for collegial support.

We suggest further research into guidance, training, and support for leaders in relational leadership and contextual understanding as these are key aspects of their role of enabling adaptive capacity in teams. Based on our findings, there is also a need for leaders to receive support for managerial tasks to free up their capacity to perform relational leadership, emphasising joint decision-making processes, values, norms, relationships, and communication practices within teams. This appears essential for nurturing organisational resilience [43] and is still an under researched area within the resilience in healthcare field. Future studies may also include questionaries to detect possible correlations between team leaders’ behaviours and how they are perceived amongst their employees, and safety improvement work [37, 44].

Finally, based on our novel findings we have developed a new framework for leadership enablers of adaptive capacity in teams (Fig. 2). By modelling leadership dynamics where key enabling factors are constantly interacting with both the local context and the relations within teams and across the local organisation, we have proposed a new theoretical contribution that supports our conceptual and practical understanding of how four key leadership enablers need to be integrated in leadership activities and approaches along with a strong relational and contextual understanding to promote adaptive capacity in hospital teams.

## Data Availability

Data retrieved from the observations and interviews are available from the corresponding author upon reasonable request and with permission from the informants.

## Abbreviations

CARE: The Concept for Applying Resilience Engineering
RN: Registered nurse
MSc: Master’s degree
PhD: Philosophiae doctor
